# Supplementary material for: Antenatal Screening for Sexually Transmitted Infections to Reduce Preterm Birth or Low Birthweight (Philani Ndiphile Study): A Randomized Three-Group Trial
Source: medRxiv. 2026 Apr 21:2026.04.15.26350805. Preprint. [Version 1] doi: 10.64898/2026.04.15.26350805 (PMC13131759; doi:10.64898/2026.04.15.26350805)
Supplement: 1 [file NIHPP2026.04.15.26350805v1-supplement-1.pdf]

Supplementary Table S1. Preterm Births by Gestational Age at Delivery

Preterm Birth Category*	All Preterm	Standard of Care	One-Time Screening	Two-Time Screening
	N = 332	n = 121	n = 118	n = 93
Extremely Preterm (< 28 weeks)	10 (3·0%)	3 (2·5%)	5 (4·2%)	2 (2·2%)
Very Preterm (28 to <32 weeks)	48 (14·4%)	18 (14·9%)	18 (15·3%)	11 (11·8%)
Moderately Preterm (32 to < 34weeks)	44 (13·3%)	16 (13·2%)	16 (13·6%)	12 (12·9%)
Late Preterm (34 to <37 weeks)	230 (69·3%)	84 (69·4%)	79 (66·9)	68 (73·1%)

*World Health Organization Classification

Supplementary Table S2A. Sensitivity analysis excluding participants with a history of preterm birth at baseline (N=1756)

Outcome	Standard of Care Risk$_0$ (R_0)	One-Time Screening Risk$_1$ (R_1)	Risk Ratio$_1$ (RR_1) R_1/R_0 (95% CI)	Two-Time Screening Risk$_2$ (R_2)	Risk Ratio$_2$ (RR_2) R_2/R_0 (95% CI)
**Primary composite***	129/581 (22·2%)	127/575 (22·1%)	0·99 (0·80–1·23)	116/600(19·3%)	0·87 (0·70–1·09)
Preterm birth (<37 weeks)	105/581 (18·1%)	104/575 (18·1%)	1·00 (0·78–1·28)	79/600 (13·2%)	0·73 (0·56–0·95)
Low birthweight (<2500 g)	71/581 (12·2%)	74/575 (12·9%)	1·04 (0·77–1·42)	74/600 (12·3%)	1·04 (0·77–1·41)

*Composite of preterm birth and/or low birthweight
Participants in the modified intention-to-treat population who reported a prior preterm birth before the index pregnancy (n = 154) were excluded. This analysis assessed whether the estimated effect of two-time screening on preterm birth was robust to exclusion of participants with a known strong baseline risk factor. Prior preterm birth history was categorized at baseline as prior preterm birth, no prior preterm birth, or nulliparous

Supplementary Table S2B. Sensitivity analysis adjusting for prior obstetric history as a covariate in the model (N=1867)

Outcome	Standard of Care Risk$_0$ (R_0)	One-Time Screening Risk$_1$ (R_1)	Risk Ratio$_1$ (RR_1) R_1/R_0 (95% CI)	Two-Time Screening Risk$_2$ (R_2)	Risk Ratio$_2$ (RR_2) R_2/R_0 (95% CI)
**Primary composite***	147/628 (23·4%)	141/611 (23·1%)	0·99 (0·81–1·21)	130/628(20·7%)	0·90 (0·73–1·11)
Preterm birth (<37 weeks)	119/628 (18·9%)	116/611 (19·0%)	1·01 (0·80–1·27)	91/628 (14·5%)	0·77 (0·61–1·00)
Low birthweight (<2500 g)	81/628 (12·9%)	87/611 (14·2%)	1·13 (0·85–1·49)	82/628 (13·1%)	1·05 (0·79–1·39)

*Composite of preterm birth and/or low birthweight
This sensitivity analysis included a three-level covariate for prior preterm and obstetric history (prior preterm birth, no prior preterm birth, nulliparous) to adjust for the baseline imbalance observed across study groups while retaining a broadly classifiable sample. Forty participants who did not report preterm birth history data at baseline and three participants with missing birthweight data were excluded from this model.

Supplementary Table S2C. Sensitivity analysis excluding nulliparous participants at baseline (N = 1197)

Outcome	Standard of Care Risk$_0$ (R_0)	One-Time Screening Risk$_1$ (R_1)	Risk Ratio$_1$ (RR_1) R_1/R_0 (95% CI)	Two-Time Screening Risk$_2$ (R_2)	Risk Ratio$_2$ (RR_2) R_2/R_0 (95% CI)
**Primary composite***	99/387 (25·6%)	91/390 (23·3%)	0·93 (0·73–1·19)	93/420 (22·1%)	0·90 (0·70–1·15)
Preterm birth (<37 weeks)	81/387 (20·9%)	83/390 (21·3%)	1·04 (0·79–1·36)	67/420 (16·0%)	0·79 (0·59–1·06)
Low birthweight (<2500 g)	50/387 (13·0%)	51/390 (13·1%)	1·05 (0·73–1·50)	54/420 (12·9%)	1·04 (0·73–1·49)

*Composite of preterm birth and/or low birthweight
This sensitivity analysis was restricted to participants in the modified intention-to-treat population who were not nulliparous; that is, had at least one prior birth before the index pregnancy.

Supplementary Table S2D. Sensitivity analysis restricting to participants with prior term births (N = 1083)

Outcome	Standard of Care Risk$_0$ (R_0)	One-Time Screening Risk$_1$ (R_1)	Risk Ratio$_1$ (RR_1) R_1/R_0 (95% CI)	Two-Time Screening Risk$_2$ (R_2)	Risk Ratio$_2$ (RR_2) R_2/R_0 (95% CI)
**Primary composite***	81/339 (23·9%)	77/353 (21·8%)	0·91 (0·69–1·20)	79/391 (20·2%)	0·85 (0·64–1·11)
Preterm birth (<37 weeks)	67/339 (19·8%)	71/353 (20·1%)	1·01 (0·75–1·37)	55/391 (14·1%)	0·71 (0·51–0·99)
Low birthweight (<2500 g)	40/339 (11·8%)	38/353 (10·8%)	0·91 (0·60–1·39)	47/391 (12·0%)	1·02 (0·69–1·51)

*Composite of preterm birth and/or low birthweight
This sensitivity analysis was restricted to participants who reported a history of prior term birth only at baseline, excluding those with any history of preterm birth and those who were nulliparous. This analysis evaluated intervention effects among participants with a pregnancy history but without prior adverse birth outcomes

Table S3. Subgroup analyses of intervention effect in participants with *C. trachomatis*, *N. gonorrhoeae*, and *T. vaginalis* at baseline

	**Standard of Care**	**One-Time Screening**	**Risk Ratio 1 (95% CI)**	**Two-Time Screening**	**Risk Ratio 2 (95% CI)**
	**(R_0)**	**(R_1)**	**(R_1)/ (R_0)**	**(R_2)**	**(R_2)/ (R_0)**
***C. trachomatis* (N = 297)**	**n = 113**	**n = 90**		**n = 94**	
Primary composite*	27 (23·9%)	20 (22·2%)	0·93 (0·56–1·55)	21 (22·3%)	0·93 (0·57–1·54)
Preterm birth	21 (18·6%)	17 (18·9%)	1·02 (0·57–1·81)	14 (14·9%)	0·80 (0·43–1·49)
Low birth weight	16 (14·2%)	14 (15·6%)	1·10 (0·57–2·13)	12 (12·8%)	0·90 (0·45–1·81)
***N. gonorrhoeae* (N = 110)**	**n = 44**	**n = 32**		**n = 34**	
Primary composite*	7 (19·4%)	9 (31·0%)	1·77 (0·74–4·25)	4 (15·4%)	0·74 (0·24–2·32)
Preterm birth	7 (15·9%)	6 (18·8%)	1·18 (0·44–3·17)	3 (8·8%)	0·55 (0·15–1·99)
Low birth weight	2 (4·5%)	7 (21·9%)	4·81 (1·07–21·65)	2 (5·9%)	1·29 (0·19–8·72)
***T. vaginalis* (N = 201)**	**n = 67**	**n = 70**		**n = 64**	
Primary composite*	19 (28·4%)	20 (28·6%)	1·01 (0·59–1·71)	11 (17·2%)	0·61(0·31–1·17)
Preterm birth	14 (20·9%)	18 (25·7%)	1·23 (0·67–2·27)	9 (14·1%)	0·67 (0·31–1·44)
Low birth weight	12 (17·9%)	12 (17·1%)	0·96 (0·46–1·98)	7 (10·9%)	0·61 (0·26–1·45)
***Any of the three STIs* (N = 507)**	**n = 180**	**n = 162**		**n = 165**	
Primary composite*	46 (25·6%)	41 (25·3%)	0·99 (0·69–1·42)	31 (18·8%)	0·74 (0·49–1·10)
Preterm birth	36 (20·0%)	34 (21·0%)	1·05 (0·69–1·59)	21 (12·7%)	0·64 (0·39–1·04)
Low birth weight	26 (14·4%)	28 (17·3%)	1·20 (0·73–1·95)	17 (10·3%)	0·71 (0·40–1·27)

*Composite of preterm birth and/or low birthweight

**Table S4**. STI Positivity at Baseline, Third Trimester, and Postnatal Visits, by Study Group			
	**Baseline (<27 weeks)**	**Third Trimester (30-34 weeks)**	**Postnatal (< 2 weeks after delivery)**
**One Time Screening**	***n = 754***		***n = 296***
C. trachomatis	112 (14·9%)	Not tested	14 (4·7%)
N. gonorrhoeae	37 (4·9%)		8 (2·7%)
T. vaginalis	81 (10·7%)		8 (2·7%)
Any of the three STIs	193 (25·6%)		25 (8·4%)
**Two Time Screening**	***n = 738***	***n=499***	***n = 328***
C. trachomatis	107 (14·5%)	24 (4·8%)	8 (2·4%)
N. gonorrhoeae	36 (4·9%)	13 (2·6%)	4 (1·2%)
T. vaginalis	71 (9·6%)	14 (2·8%)	9 (2·7%)
Any of the three STIs	186(25·2%)	46 (9·2%)	21 (6·4%)
**Standard of Care**	***n = 747***		***n = 313***
C. trachomatis	134 (17·9%)	Not tested	20 (6·4%)
N. gonorrhoeae	50 (6·7%)		10 (3·2%)
T. vaginalis	83 (11·1%)		19 (6·1%)
Any of the three STIs	218 (29·2%)		41 (13·1%)

*Baseline: enrollment at the first antenatal visit (before 27 completed weeks of gestation). Samples from participants in the Standard-of-Care Group were collected in GeneXpert transport media and tested after study completion.

†Third Trimester: Visit occurred between 30 and 34 weeks of gestation. Only participants in the Two-Time Screening Group were tested in real time; samples from the One-Time Screening and Standard-of-Care were stored and are pending testing.

‡Postnatal: Participants who attended a visit within 2 weeks of delivery were tested in all study groups
